# Complete chloroplast genome of *Erythropsis kwangsiensis* (Sterculiaceae), an endemic wild tree from South China

**DOI:** 10.1080/23802359.2019.1670111

**Published:** 2019-11-05

**Authors:** Xinmin Tian, Zezohng Qu, Rayhangul Tuerdi, Aynur Abudourexiti, Zulpenur Payzilla, Paherdin Parhat, Yongfeng Wang, Chaoyong Li, Sulayman Mamtimin

**Affiliations:** College of Life science and Technology, Xinjiang University, Urumqi, Xinjiang, China

**Keywords:** *Erythropsis kwangsiensis*, chloroplast, genome, conservation genetics

## Abstract

*Erythropsis kwangsiensis* (Sterculiaceae), a wild endangered tree that grows in South China, is an economically important species. There is scant information available on the chloroplast (cp) genome of this species. The present study is the first to analyze the cp genome of *E. kwangsiensis* using genome skimming. The whole cp genome is 160,836 bp long with 131 genes, including 84 protein-coding genes, 38 tRNA genes, and 8 rRNA genes. The GC content is 37.0%. Phylogenetic analysis revealed a close relationship to *Firmiana colorata*. This data will be useful for future investigations of conservation genetics and potential applications in breeding new varieties of this endangered and economically important tree.

*Erythropsis kwangsiensis* (Hsue), is a tree that grows in Jingxi county of south China (Wu 2007). This species has important economic value in the furniture industry and landscaping and is listed in the Chinese National Protected Plants List. However, the tree is endangered, and wild populations of *E. kwangsiensis* have decreased dramatically in recent years and only a few trees remain. Therefore, effective conservation methods are needed. Several genetic studies aimed at conservation investigated the *E. kwangsiensis* (Fan et al. 2013; Chen et al. 2014). However, the chloroplast (cp) genome has not been credibly characterized, which hinders the understanding of conservation genetics at the level of the cp genome. In this study, we sequenced, assembled and annotated the complete cp genome of *E. kwangsiensis*, based on Illumina pair-end sequencing and genome skimming methods.

Silica-dried fresh leaves of *E. kwangsiensis* were collected from Jingxi County (Guangxi, China; N 23°00′0.11″, E 106°40′0.11″). Voucher samples have been preserved in Xinjiang University (accession number TXM20190110). The traditional cetyl trimethyl ammonium bromide (CTAB) method (Doyle and Doyle 1987) was used to extract total genomic DNA. Whole-genome sequencing was performed in Suzhou, China, using an Illumina Hiseq platform. The sequencing yielded 5.0 GB of raw data. Since the raw sequence data could be mixed with non-cp DNA from the nucleus and mitochondria, we isolated the cp sequence based on the known cp genome sequences. The filtered cp sequence was used to assemble the circular cp genome using Velvet software (Zerbino and Birney 2008). Annotation analysis was performed with Plastome Annotator (Plann), which is suitable for annotation of plastomes (Huang and Cronk 2015). We generated a physical map of the genome using OGDRAW (http://ogdraw.mpimp-golm.mpg.de/) (Lohse et al. 2013). A phylogenetic maximum-likelihood (ML) tree was constructed using RaxML v.8 software (Stamatakis 2014), based on an alignment of 20 cp genomes with *Tilia amurensis* as the outgroup (Figure 1). Finally, the complete cp genome sequence together with detailed gene annotations was submitted to GenBank with the accession number MN338197 for *E. kwangsiensis.*

**Figure 1.**
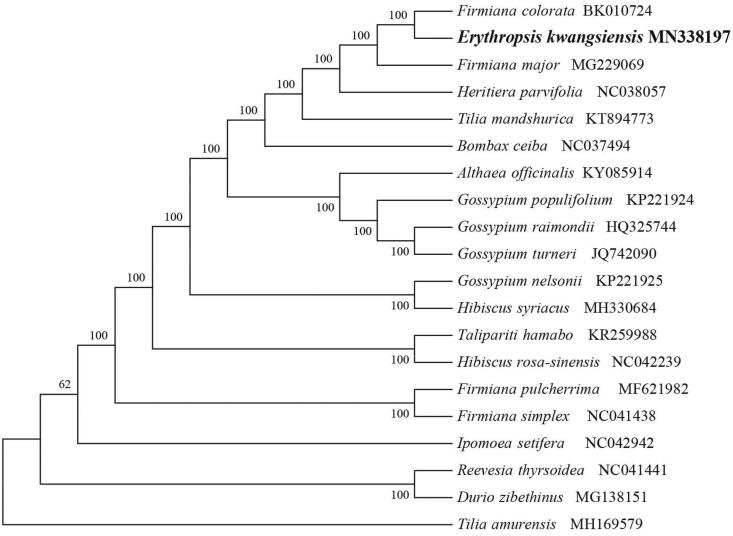
A phylogenetic tree based on 20 complete chloroplast genome sequences. Accession numbers: *Erythropsis kwangsiensis* (MN338197), *Firmiana colorata* (BK010724), *Firmiana major* (MG229069), *Heritiera parvifolia* (NC038057), *Tilia mandshurica* plastid (KT894773), *Bombax ceiba* (NC037494), *Althaea officinalis* (KY085914), *Gossypium populifolium* (KP221924), *Gossypium raimondii* (HQ325744), *Gossypium turneri* (JQ742090), *Gossypium nelsonii* (KP221925), *Hibiscus syriacus* (MH330684), *Talipariti hamabo* (KR259988), *Hibiscus rosa*-*sinensis* (NC042239), *Tilia amurensis* (MH169579), *Firmiana pulcherrima* (MF621982), *Firmiana simplex* (NC041438).

The cp genome of *E. kwangsiensis* is a typical quadripartite structure with a length of 160,836 bp, which contains inverted repeats of 25,554 bp separated by a large single-copy and small single-copy regions of 89,758 bp and 19,970 bp, respectively. The cpDNA contains 131 genes, comprising 84 protein-coding genes, 38 tRNA genes, 8 rRNA genes, and a processed pseudogene. Among the annotated genes, 15 contain one intron each (*atp*F, *clp*P, *ndh*A, *ndh*B, *pet*B, *rps*16, *rpo*C1, *rpl*16, *rp*l2, *trn*A-UGC, *trn*I-GAU, *trn*G-GCC,*trn*K-UUU, *trn*L-UAA, and *trn*V-UAC), and 2 genes (*rps*12 and *ycf*3) contain two introns each. The overall GC content of the plastome is 37.0%. Furthermore, the phylogenetic analysis of 20 plastid genomes showed that *E. kwangsiensis* was closely related to *Firmiana colorata* (Figure 1).

The data of the cp genome will be a valuable genetic resource for genetic and phylogenetic population studies and could be useful for breeding new varieties of this endangered economical tree in the future.
